# Perspectives on Systematic Analyses of Gene Function in *Arabidopsis thaliana*: New Tools, Topics and Trends

**DOI:** 10.2174/138920211794520187

**Published:** 2011-03

**Authors:** C Bolle, A Schneider, D Leister

**Affiliations:** Lehrstuhl für Molekularbiologie der Pflanzen (Botanik), Department Biologie I, Ludwig-Maximilians-Universität München, Großhaderner Str. 2, D-82152 Planegg-Martinsried, Germany

**Keywords:** *Arabidopsis thaliana*, forward genetics, gain of function, loss of function, mutant, mutation, phenotype, reverse genetics, transposon, T-DNA.

## Abstract

Since the sequencing of the nuclear genome of *Arabidopsis thaliana *ten years ago, various large-scale analyses of gene function have been performed in this model species. In particular, the availability of collections of lines harbouring random T-DNA or transposon insertions, which include mutants for almost all of the ~27,000 *A. thaliana *genes, has been crucial for the success of forward and reverse genetic approaches. In the foreseeable future, genome-wide phenotypic data from mutant analyses will become available for *Arabidopsis*, and will stimulate a flood of novel in-depth gene-function analyses. In this review, we consider the present status of resources and concepts for systematic studies of gene function in *A. thaliana*. Current perspectives on the utility of loss-of-function and gain-of-function mutants will be discussed in light of the genetic and functional redundancy of many *A. thaliana *genes.

## 1 INTRODUCTION

Because of its small size, short life cycle and compact genome, *Arabidopsis thaliana* (hereafter *Arabidopsis*) has been developed into a model system for plants. Since its nuclear genome was completely sequenced 10 years ago [1], the quest to decipher the functions of all its genes has been a major strand in *Arabidopsis *research. Forward (from-phenotype-to-gene) and reverse (from-gene-to-phenotype) genetics have been the main workhorses en route to this goal. Even before the *Arabidopsis* Genome Initiative got underway, numerous forward genetic screens had identified mutants with abnormal phenotypes that resulted in the identification and functional characterization of many genes for specific plant functions. Identification of the mutated genes is usually the most labour-intensive step in classical forward genetics. With increasing knowledge of the sequences of *Arabidopsis* genes and their transcripts, reverse genetic screens have become possible, which complement the classical approach by permitting direct analysis of mutants for specific genes, selected for their potential impact on a biological function of interest. According to the Annual Report 2010 of The Multinational Coordinated *Arabidopsis thaliana* Functional Genomics Project (http://www.arabidopsis.org/ portals/masc/masc_docs/masc_reports.jsp), the experimentally verified functions of just over 9000 of the ~27,000 *Arabidopsis* genes had been annotated as of March 2010.

*In silico* approaches can assess the functions of *Arabidopsis* genes whose homologs have been characterized in other species, or which code for proteins with conserved domains, and gene functions can be predicted by correlating data derived from functional genomics analyses, such as large-scale mRNA, protein or metabolite profiling [2-4]. However, to decipher the biological function(s) of every gene in* Arabidopsis* will require much further effort, and many projects have been initiated to create the resources required for this formidable task [5]. Technological advances in recent years have led to the assembly of large collections of *Arabidopsis* mutants bearing molecularly mapped insertions. Besides total inactivation of the gene function, some of the newly developed mutagenesis techniques allow one to alter the dosage or function of gene products, making refined analyses of gene-function relationships possible. Several databases are now available that permit researchers to scan the *Arabidopsis* genome for mutations in genes of interest and obtain the corresponding mutant lines from public stock centres.

To elucidate the function of the entire complement of *Arabidopsis* genes requires ways to systematically assess a wide range of phenotypes, which can be applied in a high-throughput manner to large numbers of mutants. Saturated collections of loss-of-function and gain-of-function lines will also be very valuable. A further challenge arises from the recognition that the vast majority of gene knock-outs in *Arabidopsis* do not give rise to obvious phenotypes, and might require functional characterisation by –omics-type analyses or the simultaneous inactivation/down-regulation of more than one gene. In this review, we summarize the state of the art in systematic gene-function analyses and highlight tools and trends in the application of systematic forward and reverse genetics to *Arabidopsis*.

## 2 TOOLS FOR THE ANALYSIS OF GENE FUNCTIONS

Several methods have been developed that enable one to change the amount or the nature of gene products either by altering the original gene through the introduction of mutations or by introducing transgenes. Since the *Arabidopsis* genome sequence was published most efforts have focused on generating loss-of-function lines for reverse genetics. In addition, methods for overexpressing genes have gained in importance more recently [6, 7].

### 2.1 Loss-of-Function Mutants (Knock-Out Approach)

The most straightforward approach to investigating the function of a gene is to characterize the phenotypic changes associated with its total inactivation [8]. Loss of gene function can be achieved by introducing point mutations or short insertions/deletions, typically by means of chemical or physical mutagenesis, or by disrupting genes by inserting larger DNA sequences, such as T-DNAs or transposable elements.

#### 2.1.1 Point Mutation and Short Insertions/Deletions

Point mutations can be introduced with high frequency by mutagenization with chemicals such as ethyl methanesulfonate (EMS), but only a small fraction of such mutations actually lead to total loss of gene function, e.g. when they generate a premature stop codon or alter functionally critical amino acids [9]. Similarly, short insertions/deletions usually eliminate gene function only if the coding sequence is affected and a frameshift results. Point mutations and short insertions/deletions have the disadvantage that the location of the mutation is random and cannot be predetermined. Therefore, in order to have a reasonable probability of finding loss-of-function mutations for a given gene, large numbers of mutants must be generated. To remove the unwanted background mutations, several rounds of backcrossing to the wild type are usually required.

The identification of individual, randomly induced point mutations or short insertions/deletions within the genome of a mutant is a laborious process involving map-based cloning [10], although recent advances in high-throughput sequencing technology [10, 11] might make resequencing of the entire mutant genome the method of choice in future. Because *Arabidopsis *reference genome sequences (such as the Col-0 ecotype) are already available, new generation sequencing of mutant genomes will be time and cost effective. Another approach to detect small changes within the genome is an adaptation of the microarray technology, which detects single nucleotide polymorphisms (SNPs) through hybridizing genomic DNA to oligonucleotides representing the entire genome spotted on chips [12]. With continuous advances in sequencing technologies, genome-based methods can replace the conventional marker-based genotyping approach. Nevertheless, because EMS-mutated plants contain many additional point mutations, it might be recommendable to narrow down first the mutation to a chromosome arm with map-based approaches and conventional techniques. 

For reverse genetic approaches employing EMS-induced mutations, the “Targeting Induced Local Lesions IN Genomes” (TILLING) method has been developed [13]. Here, the endonuclease CEL I identifies mismatches in heteroduplexes between wild-type and mutant DNA sequences after PCR-based amplification of genome sequences of interest [14]. For systematic reverse genetics screens, each target coding sequences must be covered by PCR amplicons [15]. By September 2010, 9550 TILLING lines in which the mutation has been assigned to a specific gene had been donated to public stock centres (Table **1**).

Most point mutations do not cause complete gene knock-out, but rather a decrease in gene expression (see also Section 2.2) or a change in the function of the gene product due to alterations in its sequence. Amino acid exchanges can lead to hypomorphic alleles displaying a range of phenotypes, allowing the analysis of important domains of the protein. Such variants can be pinpointed by searching the TILLING database (Table **2**). The recent release of the genome sequences of 80 different *Arabidopsis* ecotypes (with more to come when the 1001 Genomes project is completed in 2011) makes this an important tool for understanding natural variation of sequences and correlating them with evolutionary processes [16, 17] (Table **2**).

#### 2.1.2 Insertional Mutagenesis

Insertional mutagenesis has two major advantages: the mutations are labelled by the inserted fragment of known sequence (‘tag’) and insertions within the coding region have a high probability of eliminating the gene function. In *Arabidopsis*, T-DNA or transposons - mainly the *Activator* (*Ac*)/*Dissociation* (*Ds*) system - are usually employed as insertional mutagens [18]. In an international effort involving many laboratories using both systems, a large number of insertion mutants has been generated (Table **1**) which now provide nearly genome-wide coverage - about 96% of all *Arabidopsis *genes according to the Annual Report 2010 of The Multinational Coordinated *Arabidopsis thaliana* Functional Genomics Project (http://www.arabidopsis.org/portals/ masc/masc_docs/masc_reports.jsp). The regions flanking the insertions (flanking sequence tags, FST) in over 325,000 lines have been sequenced by several laboratories and mapped to the *Arabidopsis* reference genome to identify the precise insertion sites. These lines have been indexed, deposited in public stock centres and are accessible for Internet-based searches (Table **2**).

Because stock centres distribute T-DNA or transposon lines usually as populations that segregate for the mutation, a genotyping step is necessary to identify homozygous plants for the analysis of phenotypes. Recently, populations of homozygous SALK lines were generated, which can be used directly for phenotypic screens [19]. To date (September 2010) these homozygous lines represent 18,318 genes, with about 9000 genes covered by two alleles. However, a certain proportion of hemizygous lines escaped systematic genotyping, so that a certain fraction - perhaps as large as 20% - might still not be homozygous (our unpublished results). Moreover, because T-DNA lines often contain more than one insertion, for the unambiguous assignment of phenotypes to mutations either at least two insertion alleles for each gene should be analysed or the mutation be complemented with the wild-type gene.

### 2.2 Mutants with Reduced Gene Expression (Knock-Down)

The utility of loss-of-function lines is limited when mutations result in lethality, or in cases of genetic redundancy, when more than one copy of the gene exists as a result of tandem or segmental duplications or the evolution of multigene families. To overcome these drawbacks, transgene-mediated gene silencing can be used to reduce but not completely abolish (“knock-down”) the expression of gene(s) of interest. Such “knock-down” lines allow one simultaneously to down-regulate several, sequence-related genes and thus avoid the complications associated with genetic redundancy. Because knock-down lines are not null mutants and show a diverse pattern of phenotypes due to differences in the levels of activity remaining, the more subtle phenotypes induced by the partial or conditional inactivation of essential genes can also be studied.

Silencing is normally achieved by post-transcriptional down-regulation of transcript accumulation *via *small RNAs that act in a sequence-specific manner by base pairing to complementary mRNA molecules. Various strategies for small RNA-based gene silencing have been developed [20]. The first to be developed was the antisense approach, in which part of a gene was expressed in reverse orientation, usually under the control of the Cauliflower Mosaic Virus (CaMV) 35S promoter [21]. Later the RNA interference (RNAi) approach was introduced. This uses a binary hairpin RNA vector into which gene-specific tags (GSTs) are cloned [22]. Transformation of *Arabidopsis* with RNAi constructs is being systematically conducted by the AGRIKOLA (*Arabidopsis* Genomic RNAi Knock-Out Line Analysis) Consortium to create a collection of silenced *Arabidopsis* lines which are available through the Nottingham Arabidopsis Stock Centre (NASC) [23] (Tables **1** and **2**). A newer approach involves the use of artificial microRNAs (amiRNAs) [20, 24, 25]. The systematic design of amiRNA has led to the generation of 17,699 clones for use in *Arabidopsis* (as of June 2010), providing an important tool for future functional genomics studies (Table **1**). The Chimeric Repressor Silencing Technology (CRES-T) represents a novel method for creating loss-of-function mutations for the analysis of redundant plant transcription factors [26]. With CRES-T, a transcription activator of interest can be fused to a peptide or protein that converts it into a transcriptional repressor, which dominantly suppresses the expression of its target genes even in the presence of the redundant transcription factor. The phenotypic information obtained from the application of CRES-T in *Arabidopsis *is stored in the Web-based interface FioreDB [27] (Tables **1** and **2**).

### 2.3 Gain-of-Function Lines

Gain-of-function lines can be used to dissect the functions of genes that are genetically or functionally redundant, i.e. which are members of gene families or are tandemly or segmentally duplicated, or whose function can be compensated for by alternative regulatory pathways [28]. The phenotypes caused by gain-of-function mutations segregate as dominant traits [8, 29] and individual members of a gene family can produce a gain-of-function mutant phenotype that cannot be modified by other members of the family [30, 31]. Often the mutant phenotypes induced by loss-of-function and gain-of-function approaches are complementary to each other. Because of the dominant nature of the gain-of-function phenotypes, there is no need for an additional genotyping step to identify lines homozygous for the transgene.

Overexpression of a gene driven by a strong promoter can result in gain-of-function phenotypes, especially when expression is also ectopic. This strategy requires the use of full-length cDNA clones. The RIKEN *Arabidopsis* full-length (RAFL) collection now makes large numbers of such cDNA clones available. The RAFL collection was generated by inserting full-length cDNAs in the correct orientation between the CaMV 35S promoter and the NOS terminator and verifying their sequences [32]. About 10,000 different RAFL cDNA clones were then pooled and transformed into *Arabidopsis* to generate so-called ‘FOX’ (Full-length cDNA Over-eXpressing gene) lines [33]. The FOX hunting system is useful for systematic phenotypic analysis of the function of each inserted gene. Visible phenotypes were induced in about 9% of transformed lines and details of these have been collected in a database (see Section 3.2 and Table **2**).

In the activation tagging approach, DNA sequences carrying enhancers (e.g. four tandemly arranged copies of the CaMV 35S enhancer) are inserted randomly into the genome [34]. Large numbers of activation-tagging lines have been produced, and phenotypes observed could be attributed to specific genetic events by sequencing the regions flanking insertions and mapping these to the reference genome [28, 29, 34-39]. The distances between insertions and the genes affected were found to vary from 0.4 to 8.2 kb. Enhancers can control the expression of genes on both sides of the insertion and, indeed, in some cases more than one gene is affected [29, 40]. In addition to dominant gain-of-function phenotypes, knock-outs are also observed in these lines, when an insertion is located within a coding region. Between 0.1 and 1 % of the activation tagging lines showed visible phenotypes [28, 29, 35-38]. Recently, a new activation-tagging method has been developed which uses pEnLOX lines containing four tandemly repeated CaMV 35S transcriptional enhancers flanked by two *loxP* sites, and pCre lines that contain the *CRE* gene [41]. By crossing pENLOX lines to pCre lines, the CaMV 35S enhancers can be deleted, causing reversion to the wild-type phenotype, and allowing for speedier confirmation of gene-function relationships.

The Chromatin Charting Project has set itself the task of producing over 15,000 *Ds*-tagged lines of *Arabidopsis*, each of which contains within the *Ds*-tagging cassette a CaMV 35S::luciferase gene (which allows monitoring of position effects) and a *lac* operator repeat (which allows the tagged loci to be visualized by expressing fusions of fluorescent proteins with LacI within the nuclei of living plants). The selected lines will be used to evaluate transgene position effects and how these are affected by developmental or externally applied cues; since position effects on gene expression arise from alterations in chromatin architecture, this approach provides insights into global epigenetic control [42, 43] (Table **1**).

## 3 SYSTEMATIC FORWARD GENETIC SCREENS

Forward genetic screens have traditionally been used to identify genes involved in a specific biological process of interest. A range of mutant collections have been analysed, including chemically or physically mutagenized populations – which have the advantage that it is, in principle, possible to attain saturation, i.e. to generate multiple mutant alleles for each gene – and tagged mutants, which allow the mutated gene to be identified in a relatively straightforward manner. Screens for a wide variety of phenotypes have been carried out in *Arabidopsis*, and only a selection can be discussed here.

### 3.1 Generic Screens

The RIKEN Activation Tagged Lines, comprising about 50,000 independently transformed *Arabidopsis* lines [28, 40] (see Table **1**), have been screened for visually discernible phenotypes. In all, 1262 lines exhibited visible phenotypes (e.g. abnormalities in morphology, growth rate, plant coloration, flowering time or fertility) and sequence information is available for 1172 mutant loci (Table **2**). Of four dominant mutants that showed hyponastic leaves, downward-pointing flowers and decreased apical dominance, two were caused by activation of the gene *ASYMMETRIC LEAVES2* (*AS2*) and two by activation of *ASL1/LBD36* [28].

The FOX lines (see Section 2.3, Table **1**) were also screened for abnormal morphologies, fertility and leaf coloration. A total of 1487 candidate morphological mutants were found among 15,547 transformants, and of 115 pale green T_1_ mutants, 59 lines displayed the mutant phenotypes in more than 50% of the T_2_ progeny, suggesting that the phenotypes were caused by gain of function mutations (see Section 2.3). Two leaf coloration mutants were further characterized and one mutant was found to result from up-regulation of *AtPDH1 *(*Arabidospsis* prokaryotic DEVH box helicase1); knock-out of this same gene resulted in an albino phenotype [33].

### 3.2 Screens for Developmental Phenotypes

The Seed GenesProject [44-47] was designed to identify *EMBRYO DEFECTIVE *(*EMB*) genes required for embryo or seed development, and the December 2007 release of its database SeedGenes (Table **2**) includes information on 358 genes required for seed development and 605 mutant alleles with known disruptions in these genes. *EMB *genes encode proteins involved in basic cellular processes, such as DNA replication, RNA processing and protein synthesis, as well as chloroplast proteins, indicating that a functional chloroplast is needed for normal embryo development in *Arabidopsis*.

Furthermore, over 500 seedling-lethal mutants were identified in a set of 38,000 insertion mutants, of which 54 were molecularly characterized and 22 mutants were described in detail [48]. Many of the mutants displayed altered pigmentation and affect genes encoding proteins predicted to reside in the chloroplast.

To isolate mutants defective in early steps of meiotic recombination, a specialized two-step genetic screen employing 55,000 T-DNA insertion lines has been carried out [49]. In the first step, all lines were screened for fertility defects on the basis of reduced silique elongation. In the second step, differential interference contrast microscopy was used to analyse male meiotic products in developing pollen mother cells. Four genes involved in the repair of meiotic DNA double-stranded breaks were identified, together with five genes necessary for formation of meiotic DNA double-strand breaks [49].

### 3.3 Screens for Genes Important for Male or Female Gametophyte Development and Reproduction

The life cycle of plants alternates between a haploid gametophyte and a diploid sporophyte phase. Mutations that eliminate or disrupt the function of the haploid pollen (male gametophyte) or of the embryo sac (female gametophyte) can only be maintained in the heterozygous state, because such mutations cannot be transmitted through the defective gamete (reviewed in [50-52]). In addition, mutations in sporophytically expressed genes might result in male or female sterility [53, 54].

Visual inspection of siliques for 50% or more desiccated ovules, as well as screens for aberrant transmission of an antibiotic resistance marker gene, led to the identification of gametophytic mutants [55-61]. In *Arabidopsis*, due to the widely used “floral dipping” transformation protocol the primary target of T-DNA integration is the embryo sac. Therefore, it can be difficult to recover T-DNA insertional knock-out lines, in which female gametophyte function is severely compromised [56]. To overcome this problem, transposon insertion lines have been used. *Ds* transposable elements are mobilized during the sporopyhtic stage of a plant’s life cycle and most *Ds* transposon insertion lines possess further advantages like single-copy insertions without generating DNA rearrangements or truncations [62]. 24,000* Ds *insertion lines containing either a *Ds *gene trap or an enhancer trap element were screened and 130 *Arabidopsis *mutants with defects in female gametophyte development and function were identified [63]. The inheritance of the *Ds* element could be followed by the linked antibiotic resistance marker. Given the dominant mode of inheritance of the resistance phenotype, a segregation of 3:1 is expected in the progeny of a heterozygous *Ds* insertion line. Aberrant segregation ratios lower than 2:1 are often the result of a gametophytic defect. For 1.38% of the screened lines a segregation ratio of 1.5:1 or less was found and a wide variety of mutant phenotypes were observed, such as defects in different stages of embryo sac development and in processes such as pollen tube guidance, fertilization or early embryo development. Besides female gametophytic mutants, 109 lines showing predominant defects in male gametophyte transmission were also identified in this collection [64].

### 3.4 Screens for Altered Metabolism or Stress Tolerance

In a screen of the TAMARA gain-of-function collection [37] for mutants with enhanced levels of phenolic compounds, up-regulation of an R2R3-MYB transcription factor was found to be responsible for the phenotype [28, 37, 65]. Genes controlling proanthocyanidin biosynthesis have also been identified in both the TAMARA and Saskatoon collections (Table **2**) [39].

A small subset of the FOX lines has also been assessed for mutants affecting stress-inducible transcription factors. Among 43 cDNAs tested in a transgenic plant library, the authors identified salt-tolerant lines, all of which overexpressed the same bZIP-type transcription factor, AtbZIP60 [66].

The COS (controlled cDNA overexpression) system is based on a cDNA expression library that is driven by an inducible promoter. Sets of 20,000 to 40,000 transgenic seeds/seedlings were screened in three different ways, namely for ABA insensitivity, salt tolerance and for activation of a stress-responsive alcohol dehydrogenase-luciferase reporter system [67, 68]. Twenty-seven cDNAs conferring dominant, inducible stress-tolerance phenotypes were identified, and recloning of several of these cDNAs confirmed the observed phenotypes [67].

### 3.5 Chemical Genetic Screens

Treatment of mutant collection with a chemical compound to elucidate gene functions and signal transduction pathways are widely applied [69]. Limitations of this approach can be limited uptake of the compound by the plant or transport over membranes, and that possible modifications such as acetylation might inactive the compound. A prerequisite for these so called “chemical genetic screens” is the availability of a library of chemicals. The chemicals are usually used to screen for changes of a specific physiological parameter. For strigolactones, for example, that play a role during parasitic seed germination of plants such as Striga, a collection of related small molecules - cotylimides - that act as genetic suppressors of strigolactone levels were identified this way [70]. These cotylimides were used in a suppressor screen with T-DNA mutants, and light-signaling genes were identified as positive regulators of strigolactone levels [70]. Another example is the compound pyrabactin. Pyrabactin has been isolated as a synthetic seed germination inhibitor that mimics abscisic acid (ABA) in a highly selective way, thereby activating the ABA pathway. *Arabidopsis* EMS mutants were screened with pyrabactin and a new component of the ABA pathway, the* PYRABACTIN RESISTANCE 1 *(*PYR1*) gene was identified. It codes for a START protein, which was characterized as the long-elusive ABA receptor [71]. 

Besides EMS or insertion mutants multiple *Arabidopsis* natural accessions are a rich source for applying chemical genetic screens. When *Arabidopsis* natural accessions were subjected to a library of more than 10,000 compounds, twelve accession-specific molecules were identified [72]. The natural resistance to hypostatin, an inhibitor of cell expansion, was identified in this way and characterized as HYR1, a UDP glycosyltransferase.

Chemical molecules that inhibit a specific protein function result in a similar phenotype like corresponding loss-of-function mutants. Vice versa, plants treated with a compound that activates a protein function resemble gain-of-function mutants. Variation in concentration of the chemical compound can act similar to allelic series of mutations. If a chemical is able to interfere with the function of all members of a protein family, it can substitute for mutants of a higher order. The ability to perturb protein functions only in the presence of a chemical and therefore for a limited time allows the study of essential genes that would display a lethal phenotype if permanently mutated. Nevertheless, currently only a limited number of chemical compounds with a defined function are available to be applied for biological questions. Helpful are databases such as ChemMine [73] or Pubchem (http://pubchem.ncbi.nlm.nih.gov/).

### 3.6 Non-Invasive Screens

Ideally, high-throughput screens should not damage the plants investigated so that they can be used directly for the next steps in forward genetics. Besides visual screens for altered growth or coloration phenotypes, other non-invasive screening procedures have been employed. For instance, systematic measurements of chlorophyll fluorescence have been used to determine the efficiency of photosynthetic electron flow. To this end, image analysis [74, 75] and a semi-automated pulse-amplitude modulated fluorometer device [76] have been developed. Chlorophyll fluorometry has also been used for the quantitative assessment of drought survival [77]. The luciferase and green fluorescent protein (GFP; and its derivatives) reporter genes allow one to monitor changes in the expression of genes by measuring luminescence and fluorescence, respectively, and these have been utilized to identify mutants with deregulated gene expression or altered subcellular localisation of gene products (e.g. [78, 79]). Especially the luciferase screen with its very short response time can be employed to test for transient inductions. This system has been applied in stress or ABA-related mutant screens [80, 81], as well as in circadian rhythm related mutant screens [82-84]. 

Some of the lines generated within the SAIL project were transformed with a vector that carries the LAT52:GUS reporter gene, a visible cell-autonomous marker for pollen grains and tubes [44]. This population was generated in the *qrt1-2* mutant background - a mutant, which maintains the four male meiotic products in a tetrad [85] and which is very useful for non-invasive screens during male gametophyte development. Mutant lines can be classified as either homozygous or hemizygous for their T-DNA insertion by staining their pollen grains. Consequently, the *hapless* (*hap*) mutations was isolated, which impairs the development or function of haploid gametophytes [86]. 

The collection of GAL4 enhancer trap lines harbors a GAL4-responsive GFP gene as a marker to tag specific cell types and to reveal developmental transitions [87]. The lines can be screened microscopically or even viewed as images (http://www.plantsci.cam.ac.uk/Haseloff/geneControl/catalogFrame.html) and are very useful to identify enhancers for a specific cell type. The screen has been successfully applied and resulted in the isolation of Dof elements, which are involved in the regulation of guard cell gene expression [88]. In a further step some well characterized lines with specific expression patterns can be used as starter lines for EMS mutagenesis. This approach was applied to dissect the mechanisms that specify the different populations of pericycle cells. Thus, a pericycle-specific enhancer trap line was mutagenized and several mutants exhibiting qualitative or quantitative alteration of the GFP expression pattern were isolated [89].

## 4 SYSTEMATIC REVERSE GENETICS SCREENS

Efficient systematic reverse genetics requires the availability of gene-indexed mutant populations, and the use of robust, high-throughput assays for phenotypic characterizations. Studies of this type in *Arabidopsis* have benefitted from previous experience with the model organisms *Caenorhabditis* *elegans* [90] and *Saccharomyces cerevisiae* [91]. In particular, T-DNA mutant collections have been widely used for reverse genetics, as indicated by the more than 2000 cumulative citations in the literature [9]. The majority of genes in the *Arabidopsis* genome are members of gene families [1], so that redundant gene functions often have to be assessed by generating double or higher-order mutants from T-DNA lines.

In the course of systematic reverse genetic screens, lines from different collections (see Table **1**) are usually combined, in order to achieve maximum coverage of the target genes. In addition, for genes that are not covered by existing mutant collections, partial loss-of-function lines are often generated by the knock-down approach (see Section 2.2).

### 4.1 Small- and Medium-Scale Screens

Numerous small- and medium-scale reverse genetic screens have been performed which have addressed specific biological functions or multigene families. Only a small selection can be discussed here (Table **3**). One of the first systematic screens with insertion-tagged populations was performed more than 20 years ago. A collection of 8,000 *Arabidopsis* plants carrying 48,000 insertions of the maize transposable element *En-1* was screened for knock-out alleles of genes involved in flavonoid biosynthesis, utilizing a PCR-based screening protocol [92]. Another early example involved the systematic analysis of nuclear genes for photosystem I subunits, and used T-DNA or transposon insertion lines, together with knock-down lines [93, 94].

For reverse genetic analysis of the nine members of the 1-aminocyclopropane-1-carboxylate synthase (ACS) gene family, seven T-DNA mutants and two amiRNA lines were employed [95] (Table **3**). A larger screen was conducted for subtilisin-like serine proteases (subtilases), which comprises 56 members in *Arabidopsis*. Here 144 T-DNA insertion lines for 55 subtilase genes were identified. With the exception of *SDD1*, none of the lines displayed an obvious visible phenotype in the homozygous state [96]. This result highlights the need to use robust, high-throughput phenotypic screening methods to assign a phenotype, i.e. a biological function, to each gene, even in medium-scale screens.

In some cases, where genes with a known overall function were characterised by reverse genetics, an in-depth description could be provided for every member of the set. This was achieved, for instance, for all genes coding for photosystem I subunits (see above; reviewed in: [93]). The opposite case is represented by the so-called “guilt-by-association” approach, which emerged recently and is based on the concept that genes with similar functions often behave similarly with respect to their associated transcript, protein or metabolite profiles as measured by –omics technologies. Co-expression analysis combines gene expression measurements from a large number of experiments thereby providing improved statistics. Thus, co-expression analysis can be used to predict the function of genes based on the similarity of their expression patterns [97, 98].

For instance, it was shown that nuclear genes for chloroplast proteins involved in photosynthesis or plastid gene expression indeed exhibit a high level of co-expression at the transcript level [99]. This finding was then exploited to systematically characterize by reverse genetics genes of unknown function that exhibited photosynthesis gene-like transcriptional profiles, and led to the identification of PGRL1, a central component of cyclic electron flow around photosystem I (PSI) [100]. A gene for a plastidic bile-acid transporter was also isolated based on a co-expression approach [101]. Moreover, a set of genes, which are specifically expressed in pollen tubes in response to their growth in the pistil but not expressed during other stages of pollen or plant development, was identified. For 33 pollen tube–expressed genes the respective mutants were analyzed in a reverse genetics approach. The mutants were derived from a subset of the SAIL collection that was generated in the *quartet *(*qrt*) background containing a LAT52 promoter driven GUS reporter gene [44]. Indeed, seven out of 33 investigated genes were critical for pollen tube growth [102]. Other examples are reviewed in [103]. However, the “guilt-by-association” approach often involves screening large numbers of genes, and very few mutants exhibit the “desired” phenotype.

An example of chemical genetic screening in combination with reverse genetics involves *Arabidopsis* lines over-expressing 91 P450 cytochrome oxidases. In the course of this screen two P450 cytochrome oxidases that hydroxylate the therapeutic compound 8-methoxypsorelen were identified [104]. This demonstrates the potential of chemical genetic screening in combination with reverse genetics to assign specific functions to individual members of a large protein family. 

### 4.2 Large-Scale Screens

In the course of the Chloroplast 2010 Project mutations in nuclear genes that were computationally predicted to encode chloroplast-targeted proteins were subjected to a diverse set of phenotypic screens [105, 106]. During this study a total of 2733 mutants were found to be homozygous for the inspected T-DNA insertion, and 85 phenotypic characters were evaluated in each. The phenotypic tests involved quantitative measurements of metabolites, such as fatty acid methylesters in leaves or amino acid profiles in seeds, and quantification of photosynthetic parameters by a chlorophyll fluorescence assay [106]. Data are available on the project’s website (see Table **2**). Among the confirmed mutants obtained by the Chloroplast 2010 Project are mutants for At1g10310 (previously described as a pterin aldehyde reductase involved in folate metabolism) that were found to have an altered fatty acid desaturation phenotype. Single insertions in the gene *Acyl Carrier Protein4* (*ACP4*) were responsible for defects in growth, fatty acid profiles and chlorophyll fluorescence, previously associated with the knock-down of the *Acyl Carrier Protein* gene family. However, the case studies of the Chloroplast 2010 Project also stressed the difficulty of securely establishing genotype-phenotype relationships. It became clear, that - as in traditional reverse genetic screens - it is essential to confirm the genotype in advance and to exclude the possibility that a secondary mutation causes the phenotype. A second independent collection of 3246 *Ds*/*Spm*- or T-DNA-tagged homozygous lines for 1369 nuclear-encoded chloroplast proteins has been initiated [107]. Phenotypes were monitored in 3-week-old seedlings grown on agar plates and images are presented on the Chloroplast Function Database (http://rarge.psc.riken.jp/chloroplast/). 

## 5 New Trends

### 5.1 The Unimutant Collection – not Quite the Ultimate Tool?

The isolation of two homozygous insert lines for every gene in the *Arabidopsis *genome would make it possible to systematically test the phenotypic consequences of gene loss under a wide variety of conditions by forward genetics [19]. The SALK unimutant collection includes 18,318 genes, but so far only about 9000 of these are covered by two alleles. Once the unimutant collection is complete, it can be screened on several large-scale platforms for high-throughput assays e.g. [108-111]. The major advantage of screens based on the unimutant collection is obvious: exhaustive screens can be carried out with high efficiency, because loss-of-function lines for all *Arabidopsis* genes can be tested relatively quickly. But loss-of-function lines have their limitations. For instance, they provide only the first step in the analysis of essential genes (see Section 2.2). Secondly, the function of many genes will be very difficult to assess because of their apparent phenotypic silence in many assays. Thirdly, genetic redundancy will continue to hamper attempts to assign functions to members of gene families (see Section 5.2).

### 5.2 From the Unimutant to the Uni-Multimutant Collection: A Closer Approach to the Ultimate Tool

How can the problem of genetic redundancy be solved for thousands of *Arabidopsis* genes? In the simplest case, when two unlinked genes code for the same protein, the systematic generation of double mutants will make such genes manageable. In fact, in the course of the German Plant Genome Initiative (GABI), one ongoing project is dedicated to the systematic generation of segmentally duplicated genes. Segmental duplications account for the duplication of around 2900 genes in the *Arabidopsis* genome and can encompass chromosomal segments with up to about 300 genes which have been duplicated as blocks [112, 113]. In the GABI-DUPLO project, corresponding double mutants are being generated for 750 such gene pairs by crossing of T-DNA-based single mutants, selfing and PCR-based genotyping, representing a valuable complement to the unimutant collection (https://www.gabi-kat.de/duplo.html/).

Closely linked gene duplications, including tandem duplications as well as gene families containing more than two closely related genes, can only be handled by gene silencing approaches, and here the AGRIKOLA collection or amiRNA lines (see Section 2.2) promises to meet this need.

### 5.3 Targeted Mutagenesis

Site-directed mutagenesis remains a significant technical challenge in higher plants. Many attempts have been made to establish targeted mutagenesis in *Arabidopsis*, but no routine approach is available yet. Recently, zinc-finger nucleases (ZFNs) were used for targeted gene inactivation in *Arabidopsis* [114]. In this study it was shown that ZFNs engineered to act on two different genes and driven by a heat-shock promoter induced mutations in their target genes at frequencies of 3% and 2.6%, respectively.

The use of transcription activator-like effectors (TALEs) represents another approach with great potential. TALEs are injected into plant cells *via *the type III secretion pathway found in many plant pathogenic *Xanthomonas* spp. Once inside, they may contribute to disease or trigger resistance by binding to DNA and turning on TALE-specific host genes. Recent advances in understanding of the mechanistic basis of specific DNA binding by TALEs [115] should permit engineering of TALEs for arbitrary gene activation or as translational fusions for directing other proteins, such as negative regulators, methylases or nucleases, to specific DNA targets (reviewed in [116]).

### 5.4 Cataloguing of Mutant Phenotypes

Because very many research groups generate data from forward and reverse genetics experiments, a central depository for documentation of mutant phenotypes is highly desirable. To this end, The Arabidopsis Information Resource (TAIR) recently began to assign a phenotype to each mutant allele and made pictorial records of the mutant phenotypes available online (Table **2**; [117]). Curators at TAIR have been capturing phenotypic information from the *Arabidopsis* literature since 2005. As of July 2009 the TAIR database contained 7352 distinct free-text phenotypes associated with 11,381 distinct genotypes derived from almost 1500 publications.

Phenotypic databases already exist for individual mutant collections. These include, for instance, the Riken *Arabidopsis* Phenome Information Database (RAPID) (Table **2**), which is a searchable database describing morphological phenotypes of ~4,000 *Ds* transposon mutant lines [118]. Phenotypic descriptions of these lines have been classified into eight primary categories (such as seedlings, leaves, stems, flowers and siliques) and fifty secondary categories. Images of individual plants are also available and can be searched by the line number or the phenotypic categories. Similarly, the Bioassay and Phenotype Database (BAP DB) (Table **2**) is a database for exploring gene functions based on available phenotypes and for screening data from mutant, transgenic and wild-type organisms. It currently contains some project-specific phenotypic data for mutants under various abiotic stresses. BAP DB also allows users to upload their own assay and phenotypic data.

### 5.5 The Future of Systematic Phenotypic Screens

Assuming that one needs to screen a minimum of two homozygous individuals for two different mutant alleles for each of the approximately 27,000 genes in *Arabidopsis*, a total of 108,000 individual plants would have to be planted to analyse the completed unimutant collection (see Section 5.1). When grown in standard trays, a total greenhouse area of 420 m^2^ would be needed - a requirement which becomes feasible if plants are screened consecutively and not in parallel. A space-saving alternative are screens which can be performed on seedlings using either media plates or liquid assays in media-containing multi-well plates, to which different compounds to generate abiotic or biotic stresses can be easily added. Moreover, because the unimutant collection would need to be complemented with double mutants for segmentally duplicated genes (like the GABI-DUPLO lines) and RNAi lines for members of very similar members of multigene families (like AGRIKOLA or amiRNA lines), the numbers listed above might have to be increased by 25% to screen a uni-multimutant collection. Exhaustive characterisation of such populations can only be achieved by the collaborations involving many laboratories, each covering a specific set of phenotypic screens according to their specific expertise. Such screens would in many cases be repetitions of already established phenotypic assays but, because of the large number of plants to be considered, automated, innovative and non-invasive screens will have to be developed. Such screens will combine the capture of ‘classical’ visible phenotypes (such as growth rate, plant and root architecture and flowering time), specialized non-invasive assays (such as the ones to measure photosynthesis) and ‘chemical’ phenotyping (such as the profiling of metabolites, proteins and transcripts). In addition, the response of plants to different environmental conditions will have to be assessed. Finally, data collection and processing has to be streamlined to allow efficient and reliable assignment of phenotypes to individual plants. This will have to include the capability of following and documenting the fate (and phenotype) of each individual plant from sowing to disposal, preferably in an automated way. New resources for the computational analysis of images and data sets are important. In addition, standardized storage of data that allows systematic interpretation of the phenotyping results is necessary. The use of ontology terms to describe the phenotypes - like the use of Gene Ontology to describe biological processes, molecular functions and cellular components - will have to be employed. 

In spite of these many challenges, the prediction that genome-wide phenotypic data will soon become available for *Arabidopsis* does not appear to be all that far-fetched. Its realisation will no doubt stimulate a wealth of in-depth gene-function analyses.

## Figures and Tables

**Table 1:** Resources of Lines for Gene Function Analyses in *Arabidopsis*

Collection	Type	Genetic background	Number of lines	Number of insertions mapped to genome	Reference
**Loss-of-function or knock-down**					
SALK lines (SALK Institute, USA)	T-DNA	Col-0	137,259, of which 33,507 are homozygous (in progress)	166,127 (in progress)	[119]
SAIL lines (Syngenta, USA)	T-DNA	Col-0, Col-3	54,612, of which 3494 are homozygous	62,041	[120]
Wisconsin lines	Ds-Lox	Col-0	32,614	21,406	[121]
GABI-KAT lines (GABI, Germany)	T-DNA	Col-0	117,482	69,877 (in progress)	[122]
JIC SM (SLAT collection)	En/Spm	Col	~48,000; 24,000 single seed lines	26,007	[35]
CSHL-lines (Martienssen Transposon Insertion lines) (either gene or enhancer trap)	Ds-GUS	L*er*	22,084	27,398	[62, 123, 124]
RIKEN transposon-tagged lines	Ds	No-0	15,267 lines (in progress)	15,267 lines (in progress)	[118, 125-128]
INRA-Versailles lines	T-DNA with GUS	Ws	~55,000	37,142	[129, 130]
EXOTIC	Ds, Spm	L*er*, Col	23,573	in progress	[35]
AGRIKOLA	RNAi	Col	22,478 constructs, of which 2,519 are transformed into plants	-	[131]
amiRNA (CSHL-based 2010 project)	amiRNA		17,699 clones		see Table 2
Seattle *Arabidopsis *TILLING Project	EMS	Col-0 *erecta*	9,550 (in progress)		see Table 2
**gain-of-function**					
FOX hunting lines	fl cDNA over-expression	Col	5,000 (in progress) from 12,000 expected	-	[33]
Weigel lines	T-DNA_AT_	Col-7	16,398 in pools		[29]
RIKEN activation-tagged lines	T-DNA_AT_	Col	36,650 (in progress)	1,172 (in progress)	[28, 40]
SK (Saskatoon) lines	T-DNA_AT_	Col-4	~50,000 (in progress)	15,507 (in progress)	[39]
TAMARA	En/Spm_AT_	Col-0	9,471 lines	-	[37]
CRES-T	chimeric repressors		395 (in progress)	-	[132]
Chromatin charting	luciferase	Col-0	277 (in progress)	in progress	[42, 133]

Collections with less then 1000 lines are not listed unless specifically referenced in the main text. Numbers of publicly available lines have been obtained from the corresponding websites/references, the stock centers NASC, ABRC, RIKEN Bioresource center and http://signal.salk.edu/Source/AtTOME_Data_Source.html (as of September 2010). T-DNA_AT_, T-DNA activiation tagged; En/Spm_AT_, En/Spm activation-tagged.

**Table 2:** Databases/Websites for Gene Function Analyses

Resource/database	Content	Location
**Stock Centers and general information**		
*The *Arabidopsis *Information Resource (TAIR)*	Information, tools, databases	http://www.arabidopsis.org/
*Arabidopsis* Biological Resource Center (ABRC)	Information/stock centre	http://abrc.osu.edu/
European Arabidopsis *Stock Centre (NASC)*	Stock centre	http://arabidopsis.info/
Riken BioResource Center	Stock Centre (Japan)	http://www.brc.riken.go.jp/lab/epd/Eng/species/arabidopsis.shtml
Arabidopsis thaliana Resource Centre for Genomics	Databases, stock centre	http://www-ijpb.versailles.inra.fr/en/sgap/equipes/variabilite/crg/
**Collection of lines and/or phenotypes**		
SALK T-DNA collection	Information/databases on SALK T-DNA lines	http://signal.salk.edu/tabout.html
GABI-KAT lines	Information/Database for GAB-KAT lines	http://www.gabi-kat.de/
*Arabidopsis* Genetrap Website at Cold Spring Harbor Lab	GUS patterns, phenotypes and mapping data of transposon insertions	http://genetrap.cshl.edu/
RIKEN transposon- and activation-tagged lines	Information on lines and RIKEN Arabidopsis Phenome Information Database (RAPID) with phenotypes of 4000 lines	http://pfgweb.gsc.riken.go.jp/index.html http://pfgweb.gsc.riken.go.jp/pjAct.html http://www.brc.riken.jp/lab/epd/Eng/catalog/transp.shtml http://rarge.gsc.riken.go.jp/dsmutant/index.pl http://rarge.psc.riken.jp/phenome/
FOX hunting lines	Project description	http://pfgweb.gsc.riken.go.jp/pjFox.html
INRA Versailles lines	Information on INRA lines, phenotypes in Agrobact+ database	http://www-ijpb.versailles.inra.fr/en/sgap/equipes/variabilite/crg/ http://dbsgap.versailles.inra.fr/agrobactplus/English/Accueil_eng.jsp
SK (Saskatoon) lines	Project description and sequence information	http://aafc-aac.usask.ca/FST/
EXOTIC	Project description	http://www.jic.bbsrc.ac.uk/science/cdb/exotic/index.htm
Seattle *Arabidopsis *TILLING Project	Information on how to find point mutations in gene-of-interest	http://tilling.fhcrc.org/
*Arabidopsis *Genomic RNAi Knock-out Line Analysis (AGRIKOLA)	RNAi lines	http://www.agrikola.org
amiRNA (CSHL-based 2010 project)	Project description and vector information	http://2010.cshl.edu/arabidopsis/2010/scripts/main2.pl http://www.openbiosystems.com/RNAi/ArabidopsisthalianaamiRNA/
fioreDB	Project description and database	http://www.cres-t.org/index_e.html
Bioassay and Phenotype Database (BAP DB)	Information on gene functions based on phenotype and screening data	http://bioweb.ucr.edu/bapdb
**Specialized projects**		
*Seed*Gene*s* Project	List of essential genes	http://www.seedgenes.org/
*Chloroplast 2010 project*	Project description and database	http://www.plastid.msu.edu/
Chloroplast Function Database	Project description and database	http://rarge.psc.riken.jp/chloroplast/
GAL4-GFP Enhancer-trap lines	Project description, vector information and database for images	http://www.plantsci.cam.ac.uk/Haseloff/construction/catalogFrame.html
1001Genomes Project	Whole-genome sequence variation in 1001 accessions of *A. thaliana*	http://www.1001genomes.org/

**Table 3:** Selected Reverse Genetics Screens in *Arabidopsis*

Gene family / biological process	Numbers of screened genes	Reference
**Small scale screen (less than 20 genes)**		
NADP-malic enzyme / respiration	4	[134]
Sucrose synthase / carbon metabolism	6	[135]
Type A response regulator / cytokinin signaling	6 of 10	[136]
Aminocyclopropane-1-carboxylate synthase (ACS) gene family / ethylene biosynthesis	9	[95]
Photosystem I subunit (nuclear encoded) / photosynthesis	11	[93, 94]
nucleobase-ascorbate transporter / unknown biological function	12	[137]
Laccase / glyocoprotein	12 of 17	[138]
Auxin response factor / auxin-mediated transcriptional activation/repression	18 of 23	[139]
**Medium scale screens (20 to 100 genes)**		
chloroplast protein kinases and phosphatases / reversible protein phosphorylation	22	[140-142]
Pentatricopeptide repeat proteins / organelle biogenesis	39 of 450	[143]
Subtilases / serine proteases	55	[96]
pollen tube growth	33	[102]
P450 cytochrome oxidases	91	[104]
**Large-scale screens (more than 100 genes)**		
Chloroplast function	2733	[105]
Chloroplast function	1369	[107]

## References

[1] (2000). Arabidopsis Genome Initiative. Analysis of the genome sequence of the flowering plant *Arabidopsis thaliana*. Nature.

[2] Saito K, Hirai M Y, Yonekura-Sakakibara K (2008). Decoding genes with coexpression networks and metabolomics - 'majority report by precogs'. Trends Plant Sci.

[3] Allen E, Moing A, Ebbels T M, Maucourt M, Tomos A D, Rolin D, Hooks M A (2010). Correlation Network Analysis reveals a sequential reorganization of metabolic and transcriptional states during germination and gene-metabolite relationships in developing seedlings of Arabidopsis. BMC Syst. Biol.

[4] Hannah M A, Caldana C, Steinhauser D, Balbo I, Fernie A R, Willmitzer L (2010). Combined transcript and metabolite profiling of Arabidopsis grown under widely variant growth conditions facilitates the identification of novel metabolite-mediated regulation of gene expression. Plant Physiol.

[5] Alonso J M, Ecker J R (2006). Moving forward in reverse: genetic technologies to enable genome-wide phenomic screens in Arabidopsis. Nat. Rev. Genet.

[6] Ostergaard L, Yanofsky M F (2004). Establishing gene function by mutagenesis in Arabidopsis thaliana. Plant J.

[7] Hilson P (2006). Cloned sequence repertoires for small- and large-scale biology. Trends Plant Sci.

[8] Bouche N, Bouchez D (2001). Arabidopsis gene knockout: phenotypes wanted. Curr. Opin. Plant Biol.

[9] Wang Y H (2008). How effective is T-DNA insertional mutagenesis in Arabidopsis?. J. Biochem. Tech.

[10] Peters J L, Cnudde F, Gerats T (2003). Forward genetics and map-based cloning approaches. Trends Plant Sci.

[11] Schneeberger K, Ossowski S, Lanz C, Juul T, Petersen A H, Nielsen K L, Jorgensen J E, Weigel D, Andersen S U (2009). SHOREmap: simultaneous mapping and mutation identification by deep sequencing. Nat. Methods.

[12] Singer T, Fan Y, Chang H S, Zhu T, Hazen S P, Briggs S P (2006). A high-resolution map of Arabidopsis recombinant inbred lines by whole-genome exon array hybridization. PLoS Genet.

[13] Colbert T, Till B J, Tompa R, Reynolds S, Steine M N, Yeung A T, McCallum C M, Comai L, Henikoff S (2001). High-throughput screening for induced point mutations. Plant Physiol.

[14] Oleykowski C A, Bronson Mullins C R, Godwin A K, Yeung A T (1998). Mutation detection using a novel plant endonuclease. Nucleic Acids Res.

[15] Till B J, Colbert T, Tompa R, Enns L C, Codomo C A, Johnson J E, Reynolds S H, Henikoff J G, Greene E A, Steine M N, Comai L, Henikoff S (2003). High-throughput TILLING for functional genomics. Methods Mol. Biol.

[16] Ossowski S, Schneeberger K, Clark R M, Lanz C, Warthmann N, Weigel D (2008). Sequencing of natural strains of Arabidopsis thaliana with short reads. Genome Res.

[17] Weigel D, Mott R (2009). The 1001 genomes project for Arabidopsis thaliana. Genome Biol.

[18] Parinov S, Sundaresan V (2000). Functional genomics in Arabidopsis: large-scale insertional mutagenesis complements the genome sequencing project. Curr. Opin. Biotechnol.

[19] O'Malley R C, Ecker J R (2010). Linking genotype to phenotype using the Arabidopsis unimutant collection. Plant J.

[20] Ossowski S, Schwab R, Weigel D (2008). Gene silencing in plants using artificial microRNAs and other small RNAs. Plant J.

[21] Green P J, Pines O, Inouye M (1986). The role of antisense RNA in gene regulation. Annu. Rev. Biochem.

[22] Chuang C F, Meyerowitz E M (2000). Specific and heritable genetic interference by double-stranded RNA in Arabidopsis thaliana. Proc. Natl. Acad. Sci. USA.

[23] Hilson P, Allemeersch J, Altmann T, Aubourg S, Avon A, Beynon J, Bhalerao R P, Bitton F, Caboche M, Cannoot B, Chardakov V, Cognet-Holliger C, Colot V, Crowe M, Darimont C, Durinck S, Eickhoff H, de Longevialle A F, Farmer E E, Grant M, Kuiper M T, Lehrach H, Leon C, Leyva A, Lundeberg J, Lurin C, Moreau Y, Nietfeld W, Paz-Ares J, Reymond P, Rouze P, Sandberg G, Segura M D, Serizet C, Tabrett A, Taconnat L, Thareau V, Van Hummelen P, Vercruysse S, Vuylsteke M, Weingartner M, Weisbeek P J, Wirta V, Wittink F R, Zabeau M, Small I (2004). Versatile gene-specific sequence tags for Arabidopsis functional genomics: transcript profiling and reverse genetics applications. Genome Res.

[24] Schwab R, Ossowski S, Riester M, Warthmann N, Weigel D (2006). Highly specific gene silencing by artificial microRNAs in Arabidopsis. Plant Cell.

[25] Schwab R, Ossowski S, Warthmann N, Weigel D (2010). Directed gene silencing with artificial microRNAs. Methods Mol. Biol.

[26] Hiratsu K, Matsui K, Koyama T, Ohme-Takagi M (2003). Dominant repression of target genes by chimeric repressors that include the EAR motif, a repression domain, in Arabidopsis. Plant J.

[27] Mitsuda N, Hiratsu K, Todaka D, Nakashima K, Yamaguchi-Shinozaki K, Ohme-Takagi M (2006). Efficient production of male and female sterile plants by expression of a chimeric repressor in Arabidopsis and rice. Plant Biotechnol. J.

[28] Nakazawa M, Ichikawa T, Ishikawa A, Kobayashi H, Tsuhara Y, Kawashima M, Suzuki K, Muto S, Matsui M (2003). Activation tagging, a novel tool to dissect the functions of a gene family. Plant J.

[29] Weigel D, Ahn J H, Blazquez M A, Borevitz J O, Christensen S K, Fankhauser C, Ferrandiz C, Kardailsky I, Malancharuvil E J, Neff M M, Nguyen J T, Sato S, Wang Z Y, Xia Y, Dixon R A, Harrison M J, Lamb C J, Yanofsky M F, Chory J (2000). Activation tagging in Arabidopsis. Plant Physiol.

[30] Ito T, Meyerowitz E M (2000). Overexpression of a gene encoding a cytochrome P450, CYP78A9, induces large and seedless fruit in Arabidopsis. Plant Cell.

[31] Nakazawa M, Yabe N, Ichikawa T, Yamamoto Y Y, Yoshizumi T, Hasunuma K, Matsui M (2001). DFL1, an auxin-responsive GH3 gene homologue, negatively regulates shoot cell elongation and lateral root formation, and positively regulates the light response of hypocotyl length. Plant J.

[32] Seki M, Narusaka M, Kamiya A, Ishida J, Satou M, Sakurai T, Nakajima M, Enju A, Akiyama K, Oono Y, Muramatsu M, Hayashizaki Y, Kawai J, Carninci P, Itoh M, Ishii Y, Arakawa T, Shibata K, Shinagawa A, Shinozaki K (2002). Functional annotation of a full-length Arabidopsis cDNA collection. Science.

[33] Ichikawa T, Nakazawa M, Kawashima M, Iizumi H, Kuroda H, Kondou Y, Tsuhara Y, Suzuki K, Ishikawa A, Seki M, Fujita M, Motohashi R, Nagata N, Takagi T, Shinozaki K, Matsui M (2006). The FOX hunting system: an alternative gain-of-function gene hunting technique. Plant J.

[34] Tani H, Chen X, Nurmberg P, Grant J J, SantaMaria M, Chini A, Gilroy E, Birch P R, Loake G J (2004). Activation tagging in plants: a tool for gene discovery. Funct. Integr. Genomics.

[35] Tissier A F, Marillonnet S, Klimyuk V, Patel K, Torres M A, Murphy G, Jones J D (1999). Multiple independent defective suppressor-mutator transposon insertions in Arabidopsis: a tool for functional genomics. Plant Cell.

[36] Marsch-Martinez N, Greco R, Van Arkel G, Herrera-Estrella L, Pereira A (2002). Activation tagging using the En-I maize transposon system in Arabidopsis. Plant Physiol.

[37] Schneider A, Kirch T, Gigolashvili T, Mock H P, Sonnewald U, Simon R, Flugge U I, Werr W (2005). A transposon-based activation-tagging population in Arabidopsis thaliana(TAMARA) and its application in the identification of dominant developmental and metabolic mutations. FEBS Lett.

[38] Wilson K, Long D, Swinburne J, Coupland G (1996). A Dissociation insertion causes a semidominant mutation that increases expression of *TINY*, an *Arabidopsis* gene related to APETALA2. Plant Cell.

[39] Robinson S J, Tang L H, Mooney B A, McKay S J, Clarke W E, Links M G, Karcz S, Regan S, Wu Y Y, Gruber M Y, Cui D, Yu M, Parkin I A (2009). An archived activation tagged population of *Arabidopsis thaliana* to facilitate forward genetics approaches. BMC Plant Biol.

[40] Ichikawa T, Nakazawa M, Kawashima M, Muto S, Gohda K, Suzuki K, Ishikawa A, Kobayashi H, Yoshizumi T, Tsumoto Y, Tsuhara Y, Iizumi H, Goto Y, Matsui M (2003). Sequence database of 1172 T-DNA insertion sites in Arabidopsis activation-tagging lines that showed phenotypes in T1 generation. Plant J.

[41] Pogorelko G V, Fursova O V, Ogarkova O A, Tarasov V A (2008). A new technique for activation tagging in *Arabidopsis*. Gene.

[42] Lam E, Luo C, Watanabe N (2009). Charting functional and physical properties of chromatin in living cells. Curr. Opin. Genet. Dev.

[43] Rosin F M, Watanabe N, Cacas J L, Kato N, Arroyo J M, Fang Y, May B, Vaughn M, Simorowski J, Ramu U, McCombie R W, Spector D L, Martienssen R A, Lam E (2008). Genome-wide transposon tagging reveals location-dependent effects on transcription and chromatin organization in *Arabidopsis*. Plant J.

[44] McElver J, Tzafrir I, Aux G, Rogers R, Ashby C, Smith K, Thomas C, Schetter A, Zhou Q, Cushman M A, Tossberg J, Nickle T, Levin J Z, Law M, Meinke D, Patton D (2001). Insertional mutagenesis of genes required for seed development in *Arabidopsis thaliana*. Genetics.

[45] Tzafrir I, Dickerman A, Brazhnik O, Nguyen Q, McElver J, Frye C, Patton D, Meinke D (2003). The *Arabidopsis* SeedGenes Project. Nucleic Acids Res.

[46] Tzafrir I, Pena-Muralla R, Dickerman A, Berg M, Rogers R, Hutchens S, Sweeney T C, McElver J, Aux G, Patton D, Meinke D (2004). Identification of genes required for embryo development in *Arabidopsis*. Plant Physiol.

[47] Meinke D, Muralla R, Sweeney C, Dickerman A (2008). Identifying essential genes in *Arabidopsis thaliana*. Trends Plant Sci.

[48] Budziszewski G J, Lewis S P, Glover L W, Reineke J, Jones G, Ziemnik L S, Lonowski J, Nyfeler B, Aux G, Zhou Q, McElver J, Patton D A, Martienssen R, Grossniklaus U, Ma H, Law M, Levin J Z (2001). *Arabidopsis* genes essential for seedling viability: isolation of insertional mutants and molecular cloning. Genetics.

[49] De Muyt A, Pereira L, Vezon D, Chelysheva L, Gendrot G, Chambon A, Laine-Choinard S, Pelletier G, Mercier R, Nogue F, Grelon M (2009). A high throughput genetic screen identifies new early meiotic recombination functions in *Arabidopsis thaliana*. PLoS Genet.

[50] Yang W C, Sundaresan V (2000). Genetics of gametophyte biogenesis in *Arabidopsis*. Curr. Opin. Plant Biol.

[51] Drews G N, Yadegari R (2002). Development and function of the angiosperm female gametophyte. Annu. Rev. Genet.

[52] Johnson M A, Preuss D (2002). Plotting a course: multiple signals guide pollen tubes to their targets. Dev. Cell.

[53] Wilson Z A, Morroll S M, Dawson J, Swarup R, Tighe P J (2001). The *Arabidopsis MALE STERILITY1 (MS1)* gene is a transcriptional regulator of male gametogenesis, with homology to the PHD-finger family of transcription factors. Plant J.

[54] Sorensen A M, Kröber S, Unte U S, Huijser P, Dekker K, Saedler H (2003). The *Arabidopsis* ABORTED MICROSPORES (AMS) gene encodes a MYC class transcription factor. Plant J.

[55] Feldmann K A, Coury D A, Christianson M L (1997). Exceptional segregation of a selectable marker (Kan^R^) in *Arabidopsis* identifies genes important for gametophytic growth and development. Genetics.

[56] Bonhomme S, Horlow C, Vezon D, de Laissardiere S, Guyon A, Ferault M, Marchand M, Bechtold N, Pelletier G (1998). T-DNA mediated disruption of essential gametophytic genes in *Arabidopsis* is unexpectedly rare and cannot be inferred from segregation distortion alone. Mol. Gen. Genet.

[57] Christensen C A, Subramanian S, Drews G N (1998). Identification of gametophytic mutations affecting female gametophyte development in *Arabidopsis*. Dev. Biol.

[58] Howden R, Park S K, Moore J M, Orme J, Grossniklaus U, Twell D (1998). Selection of T-DNA-tagged male and female gametophytic mutants by segregation distortion in *Arabidopsis*. Genetics.

[59] Procissi A, de Laissardiere S, Ferault M, Vezon D, Pelletier G, Bonhomme S (2001). Five gametophytic mutations affecting pollen development and pollen tube growth in *Arabidopsis thaliana*. Genetics.

[60] Huck N, Moore J M, Federer M, Grossniklaus U (2003). The *Arabidopsis* mutant feronia disrupts the female gametophytic control of pollen tube reception. Development.

[61] Lalanne E, Honys D, Johnson A, Borner G H, Lilley K S, Dupree P, Grossniklaus U, Twell D (2004). *SETH1* and *SETH2*, two components of the glycosylphosphatidylinositol anchor biosynthetic pathway, are required for pollen germination and tube growth in *Arabidopsis*. Plant Cell.

[62] Sundaresan V, Springer P, Volpe T, Haward S, Jones J D, Dean C, Ma H, Martienssen R (1995). Patterns of gene action in plant development revealed by enhancer trap and gene trap transposable elements. Genes Dev.

[63] Pagnussat G C, Yu H J, Ngo Q A, Rajani S, Mayalagu S, Johnson C S, Capron A, Xie L F, Ye D, Sundaresan V (2005). Genetic and molecular identification of genes required for female gametophyte development and function in *Arabidopsis*. Development.

[64] Boavida L C, Shuai B, Yu H J, Pagnussat G C, Sundaresan V, McCormick S (2009). A collection of Ds insertional mutants associated with defects in male gametophyte development and function in *Arabidopsis thaliana*. Genetics.

[65] Gigolashvili T, Berger B, Mock H P, Müller C, Weisshaar B, Flügge U I (2007). The transcription factor HIG1/MYB51 regulates indolic glucosinolate biosynthesis in *Arabidopsis thaliana*. Plant J.

[66] Fujita M, Mizukado S, Fujita Y, Ichikawa T, Nakazawa M, Seki M, Matsui M, Yamaguchi-Shinozaki K, Shinozaki K (2007). Identification of stress-tolerance-related transcription-factor genes *via* mini-scale Full-length cDNA Over-eXpressor (FOX) gene hunting system. Biochem. Biophys. Res. Commun.

[67] Papdi C, Abraham E, Joseph M P, Popescu C, Koncz C, Szabados L (2008). Functional identification of *Arabidopsis* stress regulatory genes using the controlled cDNA overexpression system. Plant Physiol.

[68] Papdi C, Leung J, Joseph M P, Salamo I P, Szabados L (2010). Genetic screens to identify plant stress genes. Methods Mol. Biol.

[69] McCourt P, Desveaux D (2010). Plant chemical genetics. New Phytol.

[70] Tsuchiya Y, Vidaurre D, Toh S, Hanada A, Nambara E, Kamiya Y, Yamaguchi S, McCourt P (2010). A small-molecule screen identifies new functions for the plant hormone strigolactone. Nat. Chem. Biol.

[71] Park S Y, Fung P, Nishimura N, Jensen D R, Fujii H, Zhao Y, Lumba S, Santiago J, Rodrigues A, Chow T F, Alfred S E, Bonetta D, Finkelstein R, Provart N J, Desveaux D, Rodriguez P L, McCourt P, Zhu J K, Schroeder J I, Volkman B F, Cutler S R (2009). Abscisic acid inhibits type 2C protein phosphatases *via* the PYR/PYL family of START proteins. Science.

[72] Zhao Y, Chow T F, Puckrin R S, Alfred S E, Korir A K, Larive C K, Cutler S R (2007). Chemical genetic interrogation of natural variation uncovers a molecule that is glycoactivated. Nat. Chem. Biol.

[73] Girke T, Cheng L C, Raikhel N (2005). ChemMine. A compound mining database for chemical genomics. Plant Physiol.

[74] Niyogi K K, Grossman A R, Bjorkman O (1998). Arabidopsis mutants define a central role for the xanthophyll cycle in the regulation of photosynthetic energy conversion. Plant Cell.

[75] Shikanai T, Munekage Y, Shimizu K, Endo T, Hashimoto T (1999). Identification and characterization of *Arabidopsis* mutants with reduced quenching of chlorophyll fluorescence. Plant Cell Physiol.

[76] Varotto C, Pesaresi P, Maiwald D, Kurth  J, Salamini F, Leister D (2000). Identification of photosynthetic mutants of *Arabidopsis* by automatic screening for altered effective quantum yield of photosystem 2. Photosynthetica.

[77] Woo N S, Badger M R, Pogson B J (2008). A rapid, non-invasive procedure for quantitative assessment of drought survival using chlorophyll fluorescence. Plant Methods.

[78] Baruah A, Simkova K, Hincha D K, Apel K, Laloi C (2009). Modulation of O-mediated retrograde signaling by the PLEIOTROPIC RESPONSE LOCUS 1 (PRL1) protein, a central integrator of stress and energy signaling. Plant J.

[79] Tanaka H, Kitakura S, De Rycke R, De Groodt R, Friml J (2009). Fluorescence imaging-based screen identifies ARF GEF component of early endosomal trafficking. Curr. Biol.

[80] Chinnusamy V, Stevenson B, Lee B H, Zhu J K (2002). Screening for gene regulation mutants by bioluminescence imaging. Sci. STKE.

[81] Foster R, Chua N H (1999). An *Arabidopsis* mutant with deregulated ABA gene expression: implications for negative regulator function. Plant J.

[82] Southern M M, Millar A J (2005). Circadian genetics in the model higher plant, *Arabidopsis thaliana*. Methods Enzymol.

[83] Onai K, Okamoto K, Nishimoto H, Morioka C, Hirano M, Kami-Ike N, Ishiura M (2004). Large-scale screening of Arabidopsis circadian clock mutants by a high-throughput real-time bioluminescence monitoring system. Plant J.

[84] Kevei E, Gyula P, Hall A, Kozma-Bognar L, Kim W Y, Eriksson M E, Toth R, Hanano S, Feher B, Southern M M, Bastow R M, Viczian A, Hibberd V, Davis S J, Somers D E, Nagy F, Millar A J (2006). Forward genetic analysis of the circadian clock separates the multiple functions of *ZEITLUPE*. Plant Physiol.

[85] Preuss D, Rhee S Y, Davis R W (1994). Tetrad analysis possible in *Arabidopsis* with mutation of the QUARTET (QRT) genes. Science.

[86] Johnson M A, von Besser K, Zhou Q, Smith E, Aux G, Patton D, Levin J Z, Preuss D (2004). *Arabidopsis* hapless mutations define essential gametophytic functions. Genetics.

[87] Haseloff J, Dormand E L, Brand A H (1999). Live imaging with green fluorescent protein. Methods Mol. Biol.

[88] Gardner M J, Baker A J, Assie J M, Poethig R S, Haseloff J P, Webb A A (2009). GAL4 GFP enhancer trap lines for analysis of stomatal guard cell development and gene expression. J. Exp. Bot.

[89] Parizot B, Laplaze L, Ricaud L, Boucheron-Dubuisson E, Bayle V, Bonke M, De Smet I, Poethig S R, Helariutta Y, Haseloff J, Chriqui D, Beeckman T, Nussaume L (2008). Diarch symmetry of the vascular bundle in *Arabidopsis* root encompasses the pericycle and is reflected in distich lateral root initiation. Plant Physiol.

[90] Piano F, Schetter A J, Mangone M, Stein L, Kemphues K J (2000). RNAi analysis of genes expressed in the ovary of *Caenorhabditis elegans*. Curr. Biol.

[91] Giaever G, Chu A M, Ni L, Connelly C, Riles L, Veronneau S, Dow S, Lucau-Danila A, Anderson K, Andre B, Arkin A P, Astromoff A, El-Bakkoury M, Bangham R, Benito R, Brachat S, Campanaro S, Curtiss M, Davis K, Deutschbauer A, Entian K D, Flaherty P, Foury F, Garfinkel D J, Gerstein M, Gotte D, Guldener U, Hegemann J H, Hempel S, Herman Z, Jaramillo D F, Kelly D E, Kelly S L, Kotter P, LaBonte D, Lamb D C, Lan N, Liang H, Liao H, Liu L, Luo C, Lussier M, Mao R, Menard P, Ooi S L, Revuelta J L, Roberts C J, Rose M, Ross-Macdonald P, Scherens B, Schimmack G, Shafer B, Shoemaker D D, Sookhai-Mahadeo S, Storms R K, Strathern J N, Valle G, Voet M, Volckaert G, Wang C Y, Ward T R, Wilhelmy J, Winzeler E A, Yang Y, Yen G, Youngman E, Yu K, Bussey H, Boeke J D, Snyder M, Philippsen P, Davis R W, Johnston M (2002). Functional profiling of the *Saccharomyces cerevisiae genome*. Nature.

[92] Wisman E, Hartmann U, Sagasser M, Baumann E, Palme K, Hahlbrock K, Saedler H, Weisshaar B (1998). Knock-out mutants from an En-1 mutagenized *Arabidopsis thaliana* population generate phenylpropanoid biosynthesis phenotypes. Proc. Natl. Acad. Sci. USA.

[93] Jensen P E, Bassi R, Boekema E J, Dekker J P, Jansson S, Leister D, Robinson C, Scheller H V (2007). Structure, function and regulation of plant photosystem I. Biochim. Biophys. Acta.

[94] Pesaresi P, Varotto C, Richly E, Kurth J, Salamini F, Leister D (2001). Functional genomics of *Arabidopsis* photosynthesis. Plant Physiol. Biochem.

[95] Tsuchisaka A, Yu G, Jin H, Alonso J M, Ecker J R, Zhang X, Gao S, Theologis A (2009). A combinatorial interplay among the 1-aminocyclopropane-1-carboxylate isoforms regulates ethylene biosynthesis in* Arabidopsis thaliana*. Genetics.

[96] Rautengarten C, Steinhauser D, Bussis D, Stintzi A, Schaller A, Kopka J, Altmann T (2005). Inferring hypotheses on functional relationships of genes: Analysis of the *Arabidopsis thaliana* subtilase gene family. PLoS Comput. Biol.

[97] Usadel B, Obayashi T, Mutwil M, Giorgi F M, Bassel G W, Tanimoto M, Chow A, Steinhauser D, Persson S, Provart N J (2009). Co-expression tools for plant biology: opportunities for hypothesis generation and caveats. Plant Cell Environ.

[98] Loraine A (2009). Co-expression analysis of metabolic pathways in plants. Methods Mol. Biol.

[99] Biehl A, Richly E, Noutsos C, Salamini F, Leister D (2005). Analysis of 101 nuclear transcriptomes reveals 23 distinct regulons and their relationship to metabolism, chromosomal gene distribution and co-ordination of nuclear and plastid gene expression. Gene.

[100] DalCorso G, Pesaresi P, Masiero S, Aseeva E, Schunemann D, Finazzi G, Joliot P, Barbato R, Leister D (2008). A complex containing PGRL1 and PGR5 is involved in the switch between linear and cyclic electron flow in Arabidopsis. Cell.

[101] Gigolashvili T, Yatusevich R, Rollwitz I, Humphry M, Gershenzon J, Flugge U I (2009). The plastidic bile acid transporter 5 is required for the biosynthesis of methionine-derived glucosinolates in* Arabidopsis thaliana*. Plant Cell.

[102] Qin Y, Leydon A R, Manziello A, Pandey R, Mount D, Denic S, Vasic B, Johnson M A, Palanivelu R (2009). Penetration of the stigma and style elicits a novel transcriptome in pollen tubes, pointing to genes critical for growth in a pistil. PLoS Genet.

[103] Armbruster U, Pesaresi P, Pribil M, Hertle A, Leister D (2010). Update on chloroplast research: new tools, new topics and new trends. Molecular Plant.

[104] Kruse T, Ho K, Yoo H D, Johnson T, Hippely M, Park J H, Flavell R, Bobzin S (2008). In planta biocatalysis screen of P450s identifies 8-methoxypsoralen as a substrate for the CYP82C subfamily, yielding original chemical structures. Chem. Biol.

[105] Ajjawi I, Lu Y, Savage L J, Bell S M, Last R L (2010). Large-scale reverse genetics in *Arabidopsis*: case studies from the Chloroplast 2010 Project. Plant Physiol.

[106] Lu Y, Savage L J, Ajjawi I, Imre K M, Yoder D W, Benning C, Dellapenna D, Ohlrogge J B, Osteryoung K W, Weber A P, Wilkerson C G, Last R L (2008). New connections across pathways and cellular processes: industrialized mutant screening reveals novel associations between diverse phenotypes in *Arabidopsis*. Plant Physiol.

[107] Myouga F, Akiyama K, Motohashi R, Kuromori T, Ito T, Iizumi H, Ryusui R, Sakurai T, Shinozaki K (2010). The Chloroplast Function Database: a large-scale collection of *Arabidopsis* Ds/Spm- or T-DNA-tagged homozygous lines for nuclear-encoded chloroplast proteins, and their systematic phenotype analysis. Plant J.

[108] Kuromori T, Takahashi S, Kondou Y, Shinozaki K, Matsui M (2009). Phenome analysis in plant species using loss-of-function and gain-of-function mutants. Plant Cell Physiol.

[109] Finkel E (2009). With 'phenomics,' plant scientists hope to shift breeding into overdrive. Science.

[110] Yazdanbakhsh N, Fisahn J (2010). Analysis of* Arabidopsis thaliana* root growth kinetics with high temporal and spatial resolution. Ann. Bot.

[111] Joosen R V, Kodde J, Willems L A, Ligterink W, van der Plas L H, Hilhorst H W (2010). GERMINATOR: a software package for high-throughput scoring and curve fitting of *Arabidopsis* seed germination. Plant J.

[112] Blanc G, Hokamp K, Wolfe K H (2003). A recent polyploidy superimposed on older large-scale duplications in the *Arabidopsis* genome. Genome Res.

[113] Vision T J, Brown D G, Tanksley S D (2000). The origins of genomic duplications in *Arabidopsis*. Science.

[114] Osakabe K, Osakabe Y, Toki S (2010). Site-directed mutagenesis in *Arabidopsis* using custom-designed zinc finger nucleases. Proc. Natl. Acad. Sci. USA.

[115] Boch J, Scholze H, Schornack S, Landgraf A, Hahn S, Kay S, Lahaye T, Nickstadt A, Bonas U (2009). Breaking the code of DNA binding specificity of TAL-type III effectors. Science.

[116] Bogdanove A J, Schornack S, Lahaye T (2010). TAL effectors: finding plant genes for disease and defense. Curr. Opin. Plant Biol.

[117] Swarbreck D, Wilks C, Lamesch P, Berardini T Z, Garcia-Hernandez M, Foerster H, Li D, Meyer T, Muller R, Ploetz L, Radenbaugh A, Singh S, Swing V, Tissier C, Zhang P, Huala E (2008). The *Arabidopsis* Information Resource (TAIR): gene structure and function annotation. Nucleic Acids Res.

[118] Kuromori T, Wada T, Kamiya A, Yuguchi M, Yokouchi T, Imura Y, Takabe H, Sakurai T, Akiyama K, Hirayama T, Okada K, Shinozaki K (2006). A trial of phenome analysis using 4000 Ds-insertional mutants in gene-coding regions of Arabidopsis. Plant J.

[119] Alonso J M, Stepanova A N, Leisse T J, Kim C J, Chen H, Shinn P, Stevenson D K, Zimmerman J, Barajas P, Cheuk R, Gadrinab C, Heller C, Jeske A, Koesema E, Meyers C C, Parker H, Prednis L, Ansari Y, Choy N, Deen H, Geralt M, Hazari N, Hom E, Karnes M, Mulholland C, Ndubaku R, Schmidt I, Guzman P, Aguilar-Henonin L, Schmid M, Weigel D, Carter D E, Marchand T, Risseeuw E, Brogden D, Zeko A, Crosby W L, Berry C C, Ecker J R (2003). Genome-wide insertional mutagenesis of *Arabidopsis thaliana*. Science.

[120] Sessions A, Burke E, Presting G, Aux G, McElver J, Patton D, Dietrich B, Ho P, Bacwaden J, Ko C, Clarke J D, Cotton D, Bullis D, Snell J, Miguel T, Hutchison D, Kimmerly B, Mitzel T, Katagiri F, Glazebrook J, Law M, Goff S A (2002). A high-throughput *Arabidopsis* reverse genetics system. Plant Cell.

[121] Woody S T, Austin-Phillips S, Amasino R M, Krysan P J (2007). The WiscDsLox T-DNA collection: an *Arabidopsis* community resource generated by using an improved high-throughput T-DNA sequencing pipeline. J. Plant Res.

[122] Rosso M G, Li Y, Strizhov N, Reiss B, Dekker K, Weisshaar B (2003). An *Arabidopsis thaliana* T-DNA mutagenized population (GABI-Kat) for flanking sequence tag-based reverse genetics. Plant Mol. Biol.

[123] Nishal B, Tantikanjana T, Sundaresan V (2005). An inducible targeted tagging system for localized saturation mutagenesis in *Arabidopsis*. Plant Physiol.

[124] Martienssen R A (1998). Functional genomics: probing plant gene function and expression with transposons. Proc. Natl. Acad. Sci. USA.

[125] Ito T, Motohashi R, Kuromori T, Mizukado S, Sakurai T, Kanahara H, Seki M, Shinozaki K (2002). A new resource of locally transposed *Dissociation* elements for screening gene-knockout lines in silico on the *Arabidopsis* genome. Plant Physiol.

[126] Ito T, Motohashi R, Kuromori T, Noutoshi Y, Seki M, Kamiya A, Mizukado S, Sakurai T, Shinozaki K (2005). A resource of 5,814 *Dissociation* transposon-tagged and sequence-indexed lines of *Arabidopsis* transposed from start loci on chromosome 5. Plant Cell Physiol.

[127] Kuromori T, Hirayama T, Kiyosue Y, Takabe H, Mizukado S, Sakurai T, Akiyama K, Kamiya A, Ito T, Shinozaki K (2004). A collection of 11 800 single-copy Ds transposon insertion lines in *Arabidopsis*. Plant J.

[128] Sakurai T, Satou M, Akiyama K, Iida K, Seki M, Kuromori T, Ito T, Konagaya A, Toyoda T, Shinozaki K (2005). RARGE: a large-scale database of RIKEN *Arabidopsis* resources ranging from transcriptome to phenome. Nucleic Acids Res.

[129] Samson F, Brunaud V, Balzergue S, Dubreucq B, Lepiniec L, Pelletier G, Caboche M, Lecharny A (2002). FLAGdb/FST: a database of mapped flanking insertion sites (FSTs) of *Arabidopsis thaliana* T-DNA transformants. Nucleic Acids Res.

[130] Samson F, Brunaud V, Duchene S, De Oliveira Y, Caboche M, Lecharny A, Aubourg S (2004). FLAGdb++: a database for the functional analysis of the *Arabidopsis* genome. Nucleic Acids Res.

[131] Hilson P, Small I, Kuiper M T (2003). European consortia building integrated resources for *Arabidopsis* functional genomics. Curr. Opin. Plant Biol.

[132] Mitsuda N, Umemura Y, Ikeda M, Shikata M, Koyama T, Matsui K, Narumi T, Aida R, Sasaki K, Hiyama T, Higuchi Y, Ono M, Isuzugawa K, Saitoh K, Endo R, Ikeda K, Nakatsuka T, Nishihara M, Yamamura S, Yamamura T, Terakawa T, Ohtsubo N, Ohme-Takagi M (2008). FioreDB: a database of phenotypic information induced by the chimeric repressor silencing technology (CRES-T) in *Arabidopsis* and floricultural plants. Plant Biotechnol.

[133] Luo C, Lam E (2009). Chromatin charting: global mapping of epigenetic effects. Methods Mol. Biol.

[134] Wheeler M C, Tronconi M A, Drincovich M F, Andreo C S, Flügge U I, Maurino V G (2005). A comprehensive analysis of the NADP-malic enzyme gene family of *Arabidopsis*. Plant Physiol.

[135] Bieniawska Z, Paul Barratt D H, Garlick A P, Thole V, Kruger N J, Martin C, Zrenner R, Smith A M (2007). Analysis of the sucrose synthase gene family in *Arabidopsis*. Plant J.

[136] To J P, Haberer G, Ferreira F J, Deruere J, Mason M G, Schaller G E, Alonso J M, Ecker J R, Kieber J J (2004). Type-A *Arabidopsis* response regulators are partially redundant negative regulators of cytokinin signaling. Plant Cell.

[137] Maurino V G, Grube E, Zielinski J, Schild A, Fischer K, Flügge U I (2006). Identification and expression analysis of twelve members of the nucleobase-ascorbate transporter(NAT) gene family in* Arabidopsis thaliana*. Plant Cell Physiol.

[138] Cai X, Davis E J, Ballif J, Liang M, Bushman E, Haroldsen V, Torabinejad J, Wu Y (2006). Mutant identification and characterization of the laccase gene family in Arabidopsis. J. Exp. Bot.

[139] Okushima Y, Overvoorde P J, Arima K, Alonso J M, Chan A, Chang C, Ecker J R, Hughes B, Lui A, Nguyen D, Onodera C, Quach H, Smith A, Yu G, Theologis A (2005). Functional genomic analysis of the *AUXIN RESPONSE FACTOR* gene family members in *Arabidopsis thaliana*: unique and overlapping functions of ARF7 and ARF19. Plant Cell.

[140] Schliebner I, Pribil M, Zühlke J, Dietzmann A, Leister D (2008). A Survey of chloroplast protein kinases and phosphatases in *Arabidopsis thaliana*. Curr. Genomics.

[141] Bonardi V, Pesaresi P, Becker T, Schleiff E, Wagner R, Pfannschmidt T, Jahns P, Leister D (2005). Photosystem II core phosphorylation and photosynthetic acclimation require two different protein kinases. Nature.

[142] Pribil M, Pesaresi P, Hertle A, Barbato R, Leister D (2010). Role of plastid protein phosphatase TAP38 in LHCII dephosphorylation and thylakoid electron flow. PLoS Biol.

[143] Lurin C, Andres C, Aubourg S, Bellaoui M, Bitton F, Bruyere C, Caboche M, Debast C, Gualberto J, Hoffmann B, Lecharny A, Le Ret M, Martin-Magniette M L, Mireau H, Peeters N, Renou J P, Szurek B, Taconnat L, Small I (2004). Genome-wide analysis of *Arabidopsis* pentatricopeptide repeat proteins reveals their essential role in organelle biogenesis. Plant Cell.

